# Lean body weight versus total body weight to calculate the iodinated contrast media volume in abdominal CT: a randomised controlled trial

**DOI:** 10.1186/s13244-020-00920-4

**Published:** 2020-12-09

**Authors:** Moreno Zanardo, Fabio Martino Doniselli, Anastassia Esseridou, Massimiliano Agrò, Nicol Antonina Rita Panarisi, Caterina Beatrice Monti, Giovanni Di Leo, Francesco Sardanelli

**Affiliations:** 1 Department of Biomedical Sciences for Health, Università degli Studi di Milano, Via Mangiagalli 31, 20133 Milan, Italy; 2 Neuroradiology Department, Foundation IRCCS Neurological Institute “C. Besta”, Via Celoria 11, 20133 Milan, Italy; 3 Radiology Unit, IRCCS Policlinico San Donato, Via Morandi 30, 20097 San Donato Milanese, Italy; 4 Postgraduate School in Radiodiagnostics, Università degli Studi di Milano, Via Festa del Perdono 7, 20122 Milan, Italy

**Keywords:** Abdomen, Body composition, Body weight, Contrast media, Tomography (x-ray computed)

## Abstract

**Objectives:**

Iodinated contrast media (ICM) could be more appropriately dosed on patient lean body weight (LBW) than on total body weight (TBW).

**Methods:**

After Ethics Committee approval, trial registration NCT03384979, patients aged ≥ 18 years scheduled for multiphasic abdominal CT were randomised for ICM dose to LBW group (0.63 gI/kg of LBW) or TBW group (0.44 gI/kg of TBW). Abdominal 64-row CT was performed using 120 kVp, 100–200 mAs, rotation time 0.5 s, pitch 1, Iopamidol (370 mgI/mL), and flow rate 3 mL/s. Levene, Mann–Whitney *U*, and *χ*^2^ tests were used. The primary endpoint was liver contrast enhancement (LCE).

**Results:**

Of 335 enrolled patients, 17 were screening failures; 44 dropped out after randomisation; 274 patients were analysed (133 LBW group, 141 TBW group). The median age of LBW group (66 years) was slightly lower than that of TBW group (70 years). Although the median ICM-injected volume was comparable between groups, its variability was larger in the former (interquartile range 27 mL versus 21 mL, *p* = 0.01). The same was for unenhanced liver density (IQR 10 versus 7 HU) (*p* = 0.02). Median LCE was 40 (35–46) HU in the LBW group and 40 (35–44) HU in the TBW group, without significant difference for median (*p* = 0.41) and variability (*p* = 0.23). Suboptimal LCE (< 40 HU) was found in 64/133 (48%) patients in the LBW group and 69/141 (49%) in the TBW group, but no examination needed repeating.

**Conclusions:**

The calculation of the ICM volume to be administered for abdominal CT based on the LBW does not imply a more consistent LCE.

## Key points

- Calculating contrast media volume for multiphasic abdominal CT using lean body weight instead of total body weight does not reduce liver contrast enhancement (CE) variability.
- Underweight patients showed lower liver CE (median and variability).
- Further research is needed to explore such modelling to obtain a personalised approach to contrast dosing in body CT.

## Introduction

In the current clinical context, where personalised approaches to patient care have become increasingly important, research on iodinated contrast media (ICM) dose optimisation is a relevant issue [1, 2]. Individual, morphometric-tailored dosing may be at the forefront of this trend [1, 3].

Iodine concentration, injection rate, scanning delay time, saline solution flushing, patient blood pressure, and cardiac function are all factors affecting contrast enhancement (CE) in computed tomography (CT) [4–6]. Liver CE, considered the reference for parenchymal CE, is strongly influenced by ICM biodistribution into the intra- and extra-vascular space, which are both related to body size [7–9]. It is widely accepted that a larger patient needs a higher iodine load to achieve the same CE compared to a smaller patient. For this reason, dosing ICM on patient total body weight (TBW) instead of using a fixed ICM volume, which, however, is still usual practice somewhere [10], may be regarded as better [11–17].

Differently from lean body weight (LBW) in which ICM perfuses well [18, 19], adipose tissue is poorly perfused [5, 13, 20, 21]. This should be considered when studying patients with extreme body composition, such as cases with a very high/low percentage of adipose tissue (e.g. obese people versus athletes). In these patients, dosing ICM according to TBW could lead to overdosing or underdosing.

Several studies have revealed that dosing ICM based on LBW, defined as TBW minus body fat, rather than TBW could lead to a better visualisation of specific organs, blood vessels or tissues, as well as lesions or tissue anomalies [21–30]. LBW can be easily determined through a scale equipped with bioelectrical impedance analysis [31], although formulas based on patient TBW, height, and gender are available for a fast estimation [19, 32, 33].

Our hypothesis was that dosing ICM on the basis of LBW instead of TBW could avoid both overdosing obese patients and underdosing underweight patients. This should result in a more consistent ICM administration practice, with the overall net effect of a lower inter-patient variability of the liver CE. The aim of this study was to verify this hypothesis in a randomised controlled trial (RCT).

## Materials and methods

### Ethical approval

Our RCT was reported according the CONSORT statement [34]. This RCT was approved by the local Ethics Committee (IRCCS San Raffaele Hospital, authorisation number 160/int/2017) and was performed in a university hospital that is partially supported by the Italian Ministry of Health. All participants signed a written informed consent form. The trial was registered on clinicaltrials.gov as NCT03384979.

### Study design

This is a single-centre, double-blind, two-arm RCT (1:1) comparing LBW-based dosage (experimental group) versus TBW-based dosage (control group) for intravenous administration of ICM in contrast-enhanced abdominal CT. Patient enrolment started in October 2017.

### Inclusion and exclusion criteria

We enrolled patients aged ≥ 18 years referred to our Institution for a multiphasic contrast-enhanced abdominal CT for clinical indications; none of the patients underwent CT for the sole purpose of the study.

Exclusion criteria were: need for any reason of a CT protocol different from our standard protocol (e.g. tube voltage different from 120 kVp); known liver disease (cirrhosis, local or diffuse fatty infiltration, or glycogen storage disease); congestive heart failure; prior cardiac valve replacement; restrictive or constrictive pericarditis; implanted devices (pacemakers, defibrillators, insulin pumps); inability to give informed consent. Patients with liver steatosis defined as unenhanced CT values lower than 30 HU discovered after enrolment were excluded from analysis.

### Primary and secondary endpoints

The primary endpoint was liver CE. The secondary endpoints were the CT value of the descending aorta, vena cava, vena porta, and spleen; only for the descending aorta, we also calculated the CE. Secondary endpoints were considered only for exploratory analyses, and no statistical tests were performed.

### Randomisation and ICM dosages

After enrolment, patients were assigned to either the TBW or the LBW group using a random generator performed by an operator external to the clinical team. The two study groups were:

- TBW group, receiving 0.44 g iodine per kg of TBW;
- LBW group, receiving 0.63 g iodine per kg of LBW.

These two dosages were obtained on the basis of the data reported in a retrospective study on 201 patients [35]. Briefly, the former represented the mean ICM dose used in our Institution for abdominal CT, while the second was the equivalent dose expressed in terms of LBW that allowed to reach the same liver CE as that obtained using TBW.

Neither the patient or the referral radiologist was aware of which group patients were assigned to.

### LBW estimation

For each patient, TBW, height, waist circumference, and body mass index (BMI) were obtained. LBW was measured through a scale with bioelectrical impedance analysis (Tanita® mod. SC-240MA). Bioelectrical impedance analysis is a simple, quick and non-invasive technique to measure body composition. Dehghan et al. [36] demonstrated that bioelectrical impedance analysis measurements can accurately measure body fat. According to the Tanita BIA Scale specifications, measurements are within ± 5% of Underwater Weighing and DEXA (the reference standards of body composition analysis). Following the BMI international classification of the World Health Organization [37], patients were considered underweight when BMI was lower than 18.5 kg/m^2^, normal weight when from 18.5 to 25 kg/m^2^, overweight when from 25 to 30 kg/m^2^, and obese when higher than 30 kg/m^2^.

### CT protocol

All patients underwent a contrast-enhanced multiphasic CT scan of the abdomen using a 64-row unit (Somatom Definition, Siemens Healthineers) with the following technical details: tube voltage 120 kVp, tube load from 100 to 200 mAs depending on automatic exposure control system (CARE Dose 4D, Siemens Healthineers), gantry rotation time 0.5 s, pitch 1, B30f medium smooth kernel reconstruction technique.

Iopamidol (Iopamiro 370; 370 mgI/mL; Bracco Imaging SpA) was administered intravenously through a 20-gauge needle using an automatic power injector (EmpowerCTA® Contrast Injection System, Bracco Imaging SpA) at the rate of 3 mL/s, followed by 50 mL of saline solution at the same rate.

Scan delay was determined using an automated triggering hardware and a dedicated software (Bolus Tracking, Siemens Healthineers). Specifically, low-dose single-slice images were used to monitor the arrival of ICM into the descending thoracic aorta. When it enhanced over 100 HU, diagnostic scans of the abdomen were acquired after an additional average delay of 18 s for the arterial phase, 48 s for the portal venous phase, and, only in selected cases, 108 s for the nephrogenic phase. For the aim of this study, we only analysed the portal venous phase.

### Image analysis

All images were independently reviewed by a radiology resident (F.M.D.) with 4 years of experience in abdominal CT, and by a Ph.D. student (M.Z.) with 3 years of experience in image analysis. For the primary endpoint, CT attenuation measurements were obtained by manually placing one 130–170 mm^2^ region of interest in the anterior (III or IVb Couinaud) and one in the posterior (VI Couinaud) segment on the slice containing the main portal vein; these two values were averaged. Two different regions of interest were chosen to consider subtle territorial differences in liver CE. Focal hepatic lesions, blood vessels, bile ducts, calcifications, as well as artefacts, whenever present, were carefully avoided. Liver CE was calculated as the difference between the CT value measured in the portal venous phase and that measured before ICM injection. To do this, regions of interest were copy-pasted from one phase to another.

Moreover, this process was repeated for the descending aorta at the level of celiac trunk, while the spleen, vena cava, and vena porta were measured only in the portal venous phase.

### Sample size calculation

The sample size was calculated for the primary endpoint, assuming the following: alpha error 5%, statistical power 80%, and reduction in the liver CE standard deviation from 7 HU in the TBW group to 5.5 HU in the LBW group, these data deriving from a previous retrospective study [35]. A total of 274 patients (137 per group) were needed to detect such a difference using a one-sided *F* test.

### Statistical analysis

Continuous data were presented as mean and standard deviation or median and interquartile interval (or range) according to data normality, while categorical data were presented as counts and percentages. Shapiro–Wilk test was used to assess normality of distributions.

To appraise homogeneity between the two groups, preliminary comparisons were performed for baseline characteristics, including the distribution of the injected ICM volume. A *p* value of less than 0.05 [38] indicated a significant difference between the groups.

For each group, the distribution of liver CE was calculated for the whole population and for the four subgroups of BMI. Between-groups comparisons of the liver CE were performed using the one-way ANOVA or Kruskal–Wallis, depending on the distributions. Similarly, the homogeneity of variance or rank spread (the nonparametric equivalent of the variance) of liver CE was verified using the parametric or nonparametric Levene test.

Statistical analysis was performed using SPSS Statistics (SPSS v.24, IBM Inc.).

## Results

### Study population

**Fig. 1:**
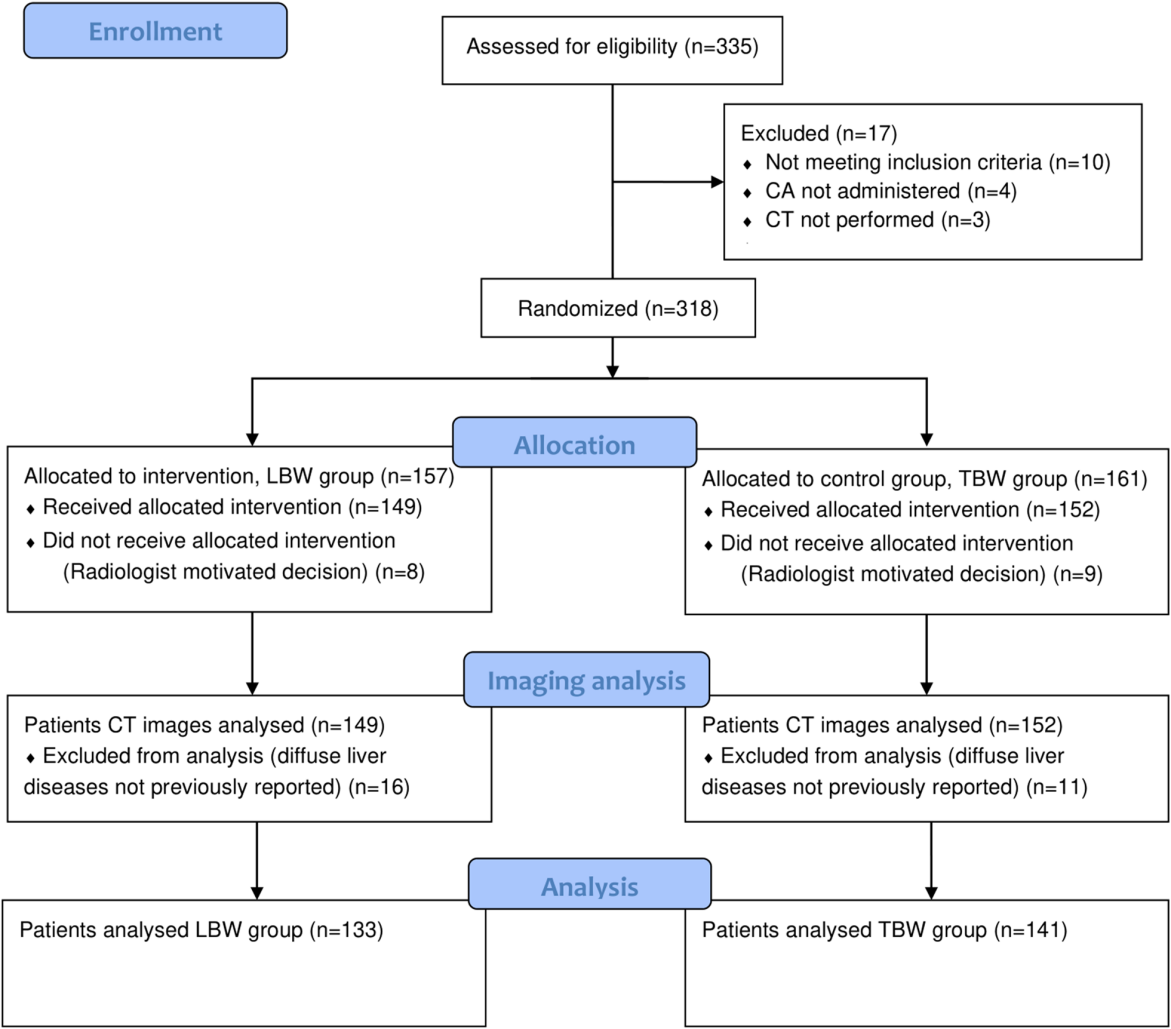
Flow diagram of the progress through the phases of two groups (enrolment, allocation, imaging analysis, and data analysis)

A total of 335 patients were enrolled from October 2017 to October 2018. Seventeen patients were excluded due to screening failure: 10 declared known liver diseases only after the informed consent was signed, while 7 did not perform the CT (*n* = 3) or performed only an unenhanced CT (*n* = 4) as per later decision of the radiologist. Thus, 318 patients were randomised and allocated to either the LBW group (*n* = 157) or the TBW group (*n* = 161). Thereafter, 8 patients randomised to the LBW group and 9 randomised to the TBW group were dropped out as the intended ICM dose was deemed too low (*n* = 10) or too high (*n* = 7) by the radiologist. Twenty-seven patients (16 patients from the LBW group and 11 from the TBW group) were excluded from analysis due to unknown diffuse liver disease (steatosis, *n* = 22; cirrhosis, *n* = 5) discovered only at CT. Thus, statistical analysis was performed on a total of 274 patients, 133 of the LBW group and 141 of the TBW group (Fig. 1).

### Patient characteristics

All distributions were non-normal at the Shapiro–Wilk test (*p* < 0.014). Thus, nonparametric statistics was used for all analyses.

The TBW group comprised 80/141 (57%) males and had a median age of 70 years (61–77), while the LBW group comprised 73/133 males (55%) and had a median age of 66 years (56–75). These and other data are reported in Table 1.

**Table 1:** Patients’ main characteristics

	TBW group (*n* = 141)	LBW group (*n* = 133)
Age (years)	70 (61–77)	66 (56–75)
Number of males	80 (57%)	73 (55%)
TBW (kg)	68 (60–77)	69 (60–77)
LBW (kg)	50 (42–58)	50 (42–58)
Height (cm)	165 (160–174)	165 (160–175)
BMI (kg/m^2^)	24 (22–28)	25 (22–27)

*TBW* total body weight, *LBW* lean body weight, *BMI* body mass index

The median ICM volume injected in the LBW group was 83 mL, comparable to that injected in the TBW group, that was 82 mL. However, the variability of this ICM volume was moderately higher in the LBW group compared to the TBW group. Specifically, the interquartile intervals were 69–96 mL (interquartile range 27 mL) and 72–93 mL (interquartile range 21 mL), respectively (*p* = 0.01).

The distribution of the unenhanced liver CT value also showed a slightly unbalanced variability between the two groups. Although the medians were 56 HU in both groups, the interquartile interval in the LBW group (51–61 HU) was moderately wider than that in the TBW group (53–60 HU) (*p* = 0.02).

### Primary and secondary endpoints

The distribution of liver CE was substantially the same in both groups. Specifically, the median liver CE was 40 (35–46) HU in the LBW group and 40 (35–44) HU in the TBW group, the difference being not significant for either the median (*p* = 0.41) or the variability (*p* = 0.23). Distributions of CT value in the three phases and the correspondent CE of the liver and aorta are reported in Table 2; this table also shows the CT value in the venous phase of cava, porta and spleen. Distributions observed in the TBW group were substantially similar to those observed in the LBW group, both in terms of medians and variability.

**Table 2:** Median and interquartile interval of CT value and CE measured in the structures analysed in the study

CT value (HU)	CE (HU)	TBW group (*n* = 141)	LBW group (*n* = 133)
Liver unenhanced		56 (53–60)	56 (51–61)
Liver arterial phase		74 (67–81)	73 (65–80)
Liver venous phase		97 (91–102)	97 (90–105)
	Liver	40 (35–44)	40 (35–46)
Aorta unenhanced		43 (40–45)	43 (40–45)
Aorta arterial phase		267 (237–305)	273 (238–304)
Aorta venous phase		125 (116–136)	125 (117–134)
	Aorta	83 (73–92)	83 (74–91)
Cava venous phase		101 (95–110)	104 (95–110)
Porta venous phase		128 (118–136)	127 (118–139)
Spleen venous phase		92 (87–98)	94 (87–99)

The median Live CE did not differ between groups at the Mann–Whitney *U* test (*p* = 0.41). Similarly, the rank spread of the liver CE did not differ between groups at the nonparametric Levene’s test (*p* = 0.23)

*HU* Hounsfield unit, *CE* contrast enhancement, *TBW* total body weight, *LBW* lean body weight

At subgroup analysis (Table 3), underweight patients of both groups showed a slightly lower liver CE together with a smaller variability compared to other BMI classes. All other BMI classes were homogeneous among them and between groups. Males and females of the TBW group had an almost identical liver CE distributions. Conversely, they were splitted in the LBW group, with males showing a higher liver CE compared to females (43 versus 37 HU) albeit with similar variability.

**Table 3:** Medians and interquartile intervals of Liver CE for subgroups of BMI and sex

	TBW group (*n* = 141)	LBW group (*n* = 133)
All patients	40 (35–44)	40 (35–46)
BMI < 18.5 kg/m^2^	36 (34–39)	37 (34–39)
BMI 18.5–25 kg/m^2^	40 (35–44)	41 (36–47)
BMI 25–30 kg/m^2^	40 (36–44)	39 (36–46)
BMI > 30 kg/m^2^	39 (35–48)	39 (35–43)
Males	40 (35–44)	43 (38–48)
Females	40 (35–46)	37 (34–42)

*TBW* total body weight, *LBW* lean body weight, *BMI* body mass index

Suboptimal liver CE (< 40 HU) was found in 64/133 (48%) patients in the LBW group and 69/141 (49%) in the TBW group, but no repeating was needed.

## Discussion

This RCT showed that dosing ICM for abdominal CT using LBW does not reduce the liver CE variability compared to a TBW-based approach. Thus, the study hypothesis was rejected.

The median injected ICM volume was the same in both groups, and no difference was found in terms of median liver CE between groups. This was expected, as the dose administered in the LBW group was retrospectively calculated so to obtain the same liver CE as in the TBW group [35]. The higher observed variability of the injected ICM volume in the LBW group compared to the TBW group was also expected, as it reflected the intrinsic higher variability of the lean mass compared to TBW in the study population. In fact, two or more persons with exactly the same TBW may show different LBW. Mathematically, the total variability is the convolution of one variability over the other.

Despite the above-mentioned observations, the liver CE distribution observed in the LBW group was substantially the same as that observed in the TBW group, including a comparable variability, as found by a similar, recently published RCT in which the variances in terms of mean hepatic CE were not significantly different for all groups (*p* > 0.05) [39]. This means that one or more hidden factors have somehow compensated by operating to an opposite trend. A potential culprit may be the liver function itself, with its capability to regulate and filter the blood flow. Another possibility is that the study hypothesis is still true, but the relation between ICM dose and liver CE is simply not linear. Moreover, these hidden compensating effects have eventually compensated also for another factor, such as the observed higher variability of the unenhanced CT value in the LBW group compared to the TBW group, that has certainly acted by increasing the liver CE variability in the LBW group. As this is a randomised trial, the only explanation that we have for this is a potential bias prompted by the exclusion of 44 patients from data statistical analysis, creating unbalances between the groups.

Other attempts were made to better personalise ICM dose by considering other indexes, such as body surface area and heart rate [40], but none of the tested parameters demonstrated any significantly better correlation with hepatic parenchymal or aortic enhancement than TBW [28]. It thus appears that liver CE originates from a nonlinear function of several variables, some of them maybe still unknown or not yet evaluated [41]. This hypothesis is also supported by subgroup analyses. In fact, obese patients of the TBW group were expected to display the highest liver CE, while data again showed some compensation effect. Underweight patients were instead partially in line with expectations, with the lowest variability but with also a slightly lower median liver CE. In practice, also subgroup analyses show a nonlinear behaviour of liver CE in relation to BMI. It is therefore clear that one single parameter, though simple to use, is not good enough for modelling the liver CE. Given the difference in liver CE observed between males and females in the LBW group, the patients’ sex ought to be considered as a potential source of variability. The analysis of the secondary endpoints seems to add nothing to the discussion.

Another result of this study is that about half of the patients had a liver CE below 40 HU, that is the threshold under which CT examinations are deemed of insufficient quality [42]. Nevertheless, none of the CT examinations was repeated, as all were considered to be diagnostic by radiologists. It is clear that the technological improvements in both hardware and software decreased not only the ionising radiation dose, but also dose of ICM needed for a diagnostic examination. It is worth mentioning that the full model-based iterative reconstruction method [43] allowed to reduce image noise by 59% and increase the signal-to-noise ratio and contrast-to-noise ratio by 144% and 166%, respectively, using a protocol with 19% reduced ICM dose. In the light of new technologies and new reconstruction algorithms that allow for ICM dose reduction, the threshold of sufficient liver CE ought to perhaps be reconsidered.

This RCT has limitations. First, although Hamer et al. [44] defined steatotic hepatitis when the liver parenchyma has an average CT value on unenhanced images lower than 40 HU, we excluded only patients with CT values below 30 HU in the unenhanced scan. Thus, low grade of steatosis has been presumably included in our study population. Second, a potential bias has been introduced, as radiologists dropped some patients from the study when the allocated ICM volume was deemed inadequate for that specific case. However, the randomisation should have harmonised the two compared groups. Finally, we did not perform a formal evaluation of image quality or grading. The fact that no CT examination was repeated does not necessarily imply diagnostic quality.

In conclusion, the calculation of ICM volume to be administered for abdominal CT based on LBW does not imply a more consistent liver CE, thus negating the study hypothesis. Liver CE appears to be a nonlinear multiparametric function of several variables that may not be modelled by simply using the TBW. Further research is needed to explore such modelling to obtain a personalised approach to contrast dosing in body CT.

## Data Availability

The datasets used and/or analysed during the current study are available from the corresponding author on reasonable request.

## Abbreviations

BMI: Body mass index
CE: Contrast-enhancement
CT: Computed tomography
HU: Hounsfield units
ICM: Iodinated contrast media
LBW: Lean body weight
RCT: Randomised controlled trial
TBW: Total body weight
